# A study on the learning experience of visitors of digital museums in STEAM education: From the perspective of visitors’ visual evaluation

**DOI:** 10.3389/fpsyg.2022.994693

**Published:** 2022-10-25

**Authors:** Xin Zhang, Jieming Hu

**Affiliations:** College of Fashion and Design, Donghua University, Shanghai, China

**Keywords:** learning experience, contemporary learning, STEAM education, m-learning, digital museum

## Abstract

Public education in museums has the interdisciplinary nature of STEAM education contemporary learning. In the contemporary learning process of the public, digital museums can rely on mobile terminals to provide people with opportunities for mobile learning. Especially since the global outbreak of COVID-19, many offline museums have been forced to close their doors or impose restrictions. How to use digital museums to better carry out the learning experience of visitors is a problem worthy of attention. Effective dissemination of visual information can help digital museums conduct educational activities more efficiently. The purpose of this study was to explore a research method for visual evaluation of visitors’ learning experiences in digital museums. The visual evaluation of visitors in the digital museum learning experience involves the layout of exhibition content, the form of exhibition space, and the way of display of exhibits. Through eye-tracking engineering experiments, we obtain and analyze the visual data of visitors during the educational experience of the digital museum to form an accurate evaluation. This method can be used to assess the visual satisfaction of visitors with the educational experience of a digital museum in mobile learning. This kind of evaluation research can provide strong data support and design optimization direction guidance for the development of digital museum public education.

## Introduction

STEAM education, characterized by interdisciplinary nature, advocates the integration of multidisciplinary knowledge to solve complex problems, so as to promote the development of students’ higher-order thinking skills (Yang and Xu, 2021). STEAM education is based on specific situations, integrates the content of various subjects, and focuses on the learning process experience (Luo and Hao, 2021). The curriculum content of STEAM education includes subject knowledge of science, technology, engineering, art, and mathematics (Yuan et al., 2020). Exploring the interdisciplinary integration of STEAM is of great benefit to the design of exhibits and the development of educational activities in museum education (Ma and Zhang, 2016). STEAM education leads students to develop discipline-integrated learning and interdisciplinary ways of thinking (Ding and Ji, 2021). The public education of museums is one of the contents with the interdisciplinary nature of STEAM education. Public education in museums involves knowledge in subjects such as art, science, technology, engineering, and mathematics. Museums are important places for people to engage in contemporary learning and informal learning experiences. Especially in the digital age, it is worth exploring how to use the resource advantages of museums to promote the improvement of STEAM education. STEAM education in museums is also more contextual and experiential. People’s experience and perception of information content shall be the main orientation of museum design (Wang and Yu, 2020). Spatial narrative provides a learning context for the development of STEAM education in museums. The theme of museum exhibition needs to construct meaning and experience discourse through spatial narrative (He and Xia, 2022). Contemporary museum exhibition is an open and dynamic communication and spread activity, the audience always in a constantly changing multimedia information Campaign (Chen, 2017). STEAM education in museums needs to provide visitors with a rich learning experience around the characteristics of museum displays. Weaving the arts together with mathematics and the sciences for museum visitors in meaningful ways is currently an area of development for many museum professionals (Saraniero and Kelton, 2019). There are many STEAM educational activities in the museum. The National World War II Museum in the United States, the CDC Museum, and the Queensland Museum in Australia have all carried out courses related to STEAM education (Zhang et al., 2022). STEAM education in the museum revolves around multi-dimensional display content such as collections and spaces. The collection is based on the STEAM education concept from the presentation of “cultural relics” to the display and restoration of “space” situation, and finally enables participants to acquire the ability to think and solve problems independently (Cheng et al., 2022). The subject information of the display, the spatial information of the display and the information of the exhibits together contribute to the main body of these information. These multi-dimensional information provide visitors with a multi-dimensional information experience. Museums’ visitors can play an active role both during and after the visit that will allow them to shape a significant experience (Carmen et al., 2018). In the process of public contemporary learning, digital museums can rely on mobile terminals to provide people with opportunities for mobile learning. The virtual museum is a virtual display of the traditional physical museum through three-dimensional modeling and virtual reality technology (Meng and Liu, 2015). The construction of online virtual experience hall is also an important measure for the dissemination of museum culture and education (Rao and Li, 2021). Conceptually, the concept of digital museums is broader. But they are all digital means to provide visitors with a virtual experience that is different from a physical museum. Digital museums provide visitors by virtualizing space, exhibits, and information. The digital museum experience is not limited by time and space. The physical museum is to display the physical exhibits in the real space. For general museums, a digital museum is a digital representation of a physical museum. The physical museum provides the basis for the construction of the digital museum. However, physical museums are more susceptible to factors such as time and space. Especially since the global outbreak of COVID-19, many offline museums have been forced to close their doors or impose restrictions. How to make better use of digital museums to carry out educational experience is an issue worthy of attention. When the digitalization of museums has become an inevitable development trend, it is particularly important and urgent to redesign more humanized and intelligent services and experiences (Zhang et al., 2015). Digital museums should provide visitors with a multi-dimensional learning experience during the construction process.

Visual evaluation is very important for the effective development of STEAM education in digital museums. The application of eye tracking for the evaluation of humans’ viewing behavior is a common approach in psychological research (Kurzhals et al., 2016). The gaze data of visitors in museums can reflect the interests and needs of visitors (Yi et al., 2020). The visitor’s visual evaluation mentioned in this study refers to the visual reflection produced by the visitor when viewing the digital museum using a terminal such as a computer. These visual evaluation contents originate from the exhibition content, spatial form, and display method of the digital museum. The purpose of the word museum is to experience the exhibitions of offline museums online through the Internet without being limited by time and space. Digital museum has a variety of media forms which can mobilize the audience to participate through the virtual reality and interactive media (Chang and Rong, 2011). At present, most online digital museums are built on the basis of offline physical museums. With the continuous development of digital twin technology, the digital museum can become the test body of the physical museum, which can help the physical museum to complete simulation experiments and performance tests under various hypothetical conditions. The digital twin is not only a virtual online of real entities, but also simulates the behavior of objects in the real environment (Wang et al., 2021). This will provide predictions and a virtual simulation experience for the development of STEAM education in the museum. Since the digital museum is a twin copy of the physical museum, the two have a mapping relationship in the content layout of the exhibition, the spatial scene displayed, and the display method of the exhibits. Constantly testing and improving the narrative of museum exhibitions through timely feedback from different audiences can make museum curation more scientific (Li, 2020). Each space uses different display techniques and narrative methods when displaying different exhibits, and sets different plot contents to make the space prioritized, which in turn affects the emotional feelings of the visitors (Zhang, 2020). The exhibits determine the content of the story, the curator digs the direction of the story, the design shapes the expression of the story, and the audience influences the completion of the story (Hou, 2019). The process of the story is used as the basis for the floor plan, as well as the script for the audience’s tour route (Li, 2014). It can be seen that the narrative, space and exhibits in the museum are important and very profound for the learning experience of visitors.

In addition, the development of STEAM education in the museum space needs to allow visitors to clearly understand the exhibition content layout of the exhibition, because it directly interprets the spatial story of the exhibition. The shaping of the design and the formation of the narrative atmosphere in the space depend on the spatial form of the display. The way the exhibits are displayed is the most basic expression. Therefore, the visual evaluation of visitors in the educational experience of the digital museum involves the layout of exhibition content, the form of exhibition space, and the way the exhibits are displayed. These contents are also the visual evaluation contents that visitors need to focus on in the STEAM educational experience of the digital museum. Visitor research employs a range of methods to assess whether exhibits attract and hold people’s attention and to explore people’s experience and understanding of exhibitions (vom Lehn, 2006). We can obtain and analyze the visual data of visitors in the process of digital museum education experience to form accurate evaluation. This method can be used to assess the visual satisfaction of visitors with the digital museum learning experience in mobile learning. This kind of evaluation research can provide strong data support and design optimization direction guidance for the development of STEAM education in digital museums.

## Data visualization analysis of related research

The evaluation research of the visitor’s learning experience of digital museums in STEAM education needs to have an international perspective. We carry out quantitative analysis by visualizing the literature. We selected the Web of Science database and searched “Digital Museum” and “Visitor” as the subjects to search. The information we retrieved is set to be published from 2016 to 2021, and a total of 179 related journals and papers were retrieved. By importing the CiteSpace bibliometric analysis tool, we eliminated duplicates and screened the imported data, and there were 171 related literature information left. We implement visual analysis of researchers, research institutions, and keywords through CiteSpace. Through this method, we sort out and summarize the research hotspots and patterns of digital museums and visitors.

According to the researcher’s knowledge map drawn by CiteSpace and related literature analysis, it is found that the researchers based on the digital museum and visitors are mainly personnel of various research institutions, university faculty and postgraduates. According to the knowledge graph, under the premise that the threshold is 1, a total of 163 nodes are generated. The figure shows a total of 114 connections, and the density is only 0.0086. This suggests a low and loose distribution among investigators. The research frequency of each researcher is also low, the highest is only 3, and the centrality is low. From the map, the researcher does not form a clear cluster.

**FIGURE 1:**
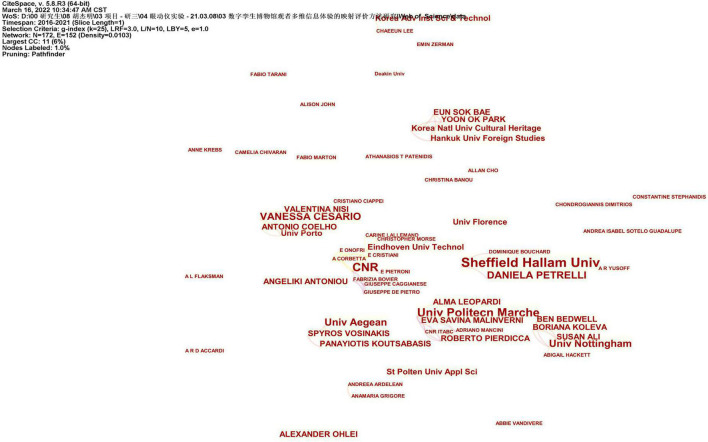
Knowledge graph of relevant research institutions. Image source: The information was retrieved by our research team and exported after CiteSpace analysis. Data source: The authors were retrieved through Web of Science.

According to the knowledge map of research institutions drawn by CiteSpace (Figure 1) and related background data analysis, it is found that the research institutions for digital museums and virtual museums are mainly concentrated in European and American universities and research institutes. In the picture, Sheffield Hallam Univ (Sheffield Hallam University) appears six times, Univ Aegean (Aegean University) appears five times, CNR (National Research Council) appears four times, and Univ Politics Marche (Marche University of Political Science) appears four times, MIT (Massachusetts Institute of Technology) appeared three times. On the whole, although the research universities have a certain authority, the research frequency is generally low. The research among the various research institutions is not very closely linked, and the research force is relatively scattered. According to what is known in the knowledge graph, 163 nodes and 114 connections are displayed, and the density is only 0.0086. This shows that the distribution among the overall research institutions is scattered, and the research direction has not converged. Therefore, it is necessary to deepen the research scope and research level of the current digital museum.

**FIGURE 2:**
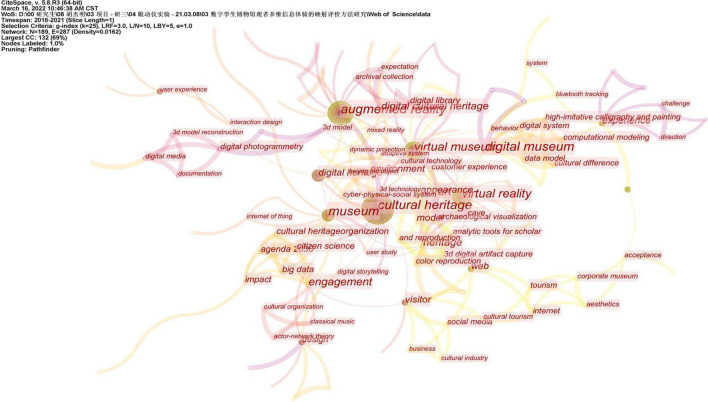
Keyword o-occurrence network graph. Image source: The information was retrieved by our research team and exported after CiteSpace analysis. Data source: The authors were retrieved through Web of Science.

Through CiteSpace to draw the relevant keyword knowledge map, the co-occurrence analysis and cluster analysis of the keywords are realized. This method summarizes the current hotspots and trends in visitor research in digital museums. We set the node type to keyword in CiteSpace, set “Time Slicing” to “2016–2021,” and set the time slice to 1 for analysis. However, due to the limited number of documents in the study of digital museums, the threshold was set to 1 to obtain the co-occurrence network map of keywords (Figure 2). From the perspective of the co-occurrence frequency of keywords, the literature research mainly focuses on the following keywords: cultural heritage, augmented reality, virtual reality, virtual museum, museum, digital heritage, digital museum, heritage, and design. Among them, the key word “cultural heritage” has the most intensive collinear network, with a total frequency of 26 times. Other major keywords appear as follows: “augmented reality” 18 times, “virtual reality” 17 times, and “virtual museum” 13 times. From the co-occurrence map of keywords, we can find that the main research content at present is “cultural heritage” as the core. The application of technologies such as virtual reality and augmented reality to museum displays is also a research focus. The current related research regards digital museums as an extension of the functions of physical museums. The study of visitors’ learning experience and visual evaluation in digital museums has not been paid enough attention, and in-depth research and theoretical system construction are still needed.

**FIGURE 3:**
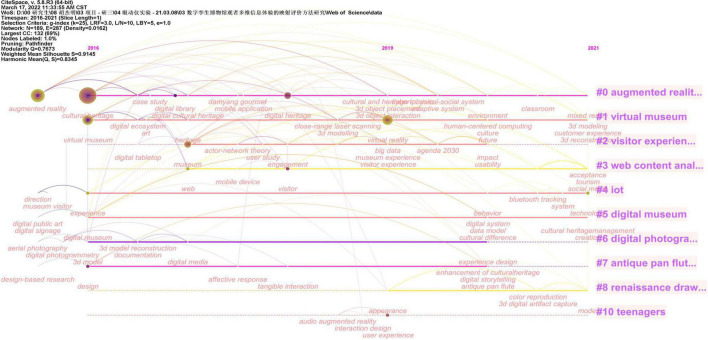
Keyword cluster visualization graph. Image source: The information was retrieved by our research team and exported after CiteSpace analysis. Data source: The authors were retrieved through Web of Science.

Keyword clustering reflects the change analysis of literature in a certain research field within a certain period of time. We set the node type to keyword in CiteSpace, set the time slice to 1, and set the visualization to “Timeline Show.” We present keyword clusters and hot topics as a visualization in chronological order (Figure 3). In the end, 10 cluster labels were formed, from high to low, they were “Augmented Reality,” “Virtual Museum,” “Visitor Experience,” “Interface Content Analysis,” “Internet of Things,” “Digital Museum,” “Digitizing Images,” etc. The keyword clustering map drawn by CiteSpace can extract the research hotspots of “Digital Museum” and “Visitor.” By setting the threshold top *N* of each time slice to 50, cluster analysis, keyword selection, and cluster extraction of LLR algorithm are performed. We obtained the keyword clustering of digital museum-related research (Table 1). The construction of a digital museum is inseparable from the support of technology, and virtual reality technology is a key technical application in the process of museum informatization. According to the top five of the cluster tags, we can find that the association of tag 0 “augmented reality” is mainly in augmented reality, digital cultural heritage, digital library, etc., that is, at the level of digital museum construction. In addition to the virtual museum itself, the association of label 1 “virtual museum” also includes human-computer interaction, that is, at the interactive level of the virtual museum. The association of label 2 “Visitor Experience” mainly focuses on the visitor experience and the museum experience, that is, at the level of the visitor’s experience. Label 3 “Interface Content Analysis” is related to commercial museums, digitization, and web pages, that is, at the level of web interface display. Label 4 “Internet of Things” is mainly related to the Internet of Things, museum optimization, museum simulators, and digital usage guides, that is, at the application level of the digital Internet of Things. Label 5 “Digital Museum” is mainly related to social media marketing, three-dimensional display, data model, etc., that is, at the operational level of digital museums. The protection of cultural heritage and augmented reality technology are the hotspots of digital museum research. Although visitor experience has received attention in digital museum research, there has been insufficient attention in research on visitor learning experience. There is insufficient research on the association between visitor learning experience and STEAM education. With the continuous improvement of the virtual technology and web content construction of the digital museum, the research on the learning experience of the digital museum based on the visual evaluation of visitors is particularly important.

**TABLE 1:** Keyword clustering table of related research (the table information is processed by CiteSpace after our research team retrieves the information).

Cluster number	Label	Number of documents	Average year	LLR log-likelihood label words
0	Augmented reality	29	2018	Augmented reality (15.3, 1.0E−4); digital cultural heritage (7.56, 0.01); digital heritage (7.56, 0.01); digital libraries (5.02, 0.05); cultural heritage (3.78, 0.1)
1	Virtual museum	23	2018	Virtual museum (9.16, 0.005); virtual museums (6.08, 0.05); human computer interaction (hci) (6.08, 0.05); interaction paradigms (6.08, 0.05); human-centered computing (6.08, 0.05)
2	Visitor experience	21	2018	Visitor experience (4.19, 0.05); museum experience (4.19, 0.05); interpretation (4.19, 0.05); open innovation (4.19, 0.05); managerialization (4.19, 0.05)
3	Web content analysis	13	2020	Web content analysis (5.61, 0.05); corporate museums (5.61, 0.05); digitalization (5.61, 0.05); e-commerce (5.61, 0.05); website (5.61, 0.05)
4	IoT	11	2018	IoT (11.09, 0.001); museum optimization (5.49, 0.05); museum knowledge (5.49, 0.05); museum simulator (5.49, 0.05); use of digital guides (5.49, 0.05)
5	Digital museum	10	2019	Digital museum (5.79, 0.05); social media marketing (5.49, 0.05); three-dimensional displays (5.49, 0.05); data models (5.49, 0.05); museum marketing (5.49, 0.05)

## Related experimental materials and procedures

This research uses the roaming and scene screenshots of the online digital museum exhibition (Nanjing Museum – Yutang Jiaji Exhibition) as experimental stimulation materials. Yutang Fine Artifacts Exhibition is a permanent exhibition open to the public in Nanjing Municipal Museum. The content of the exhibition is divided into 11 themed exhibition areas. The content layout of the exhibition is classified and arranged according to the cultural relic properties of the exhibits. For example, the “Hairpin Ying Hall” is themed with the ornaments and wine utensils of the nobles of the Ming Dynasty. The “calligraphy and painting small building” mainly focuses on calligraphy and painting exhibits. The “pot seclusion” mainly focuses on the exhibits of purple clay pots. The “Qing Hui Pavilion” mainly displays Guqin exhibits. Cultural relics with a longer history are arranged in the front exhibition hall. The cultural relics exhibits in the Ming and Qing dynasties are not completely arranged according to the chronological clues. The spatial form of the exhibition is mainly based on the traditional Chinese Jiangnan ancient architectural style. Each exhibition hall is independent and is mainly arranged in a relatively compact linear arrangement. The color palette of this museum space is dominated by the warm red of mahogany and the warm yellow of imitation candlelight. The exhibition space is also equipped with plaques, Chinese grilles, Chinese circular window holes, and Chinese garden corridors. The exhibition hall space is separated by designing doors, windows, grilles, and small scenes, which have the characteristics of traditional Chinese gardens. The exhibits in the exhibition include a total of 30 cultural relics such as bronze ware, porcelain, lacquer ware, purple clay pot, calligraphy, and painting works. Exhibits are displayed in display cabinets made in the style of Ming-style furniture. The title and description board of the exhibition hall are displayed in the form of plaques and hanging screens respectively. The online digital exhibition hall of Yutang Jiaji Exhibition is free and open to the public. This digital exhibition can be browsed through a mobile terminal or a computer. This digital exhibition uses WEB3D technology to digitally replicate the offline physical museum exhibition. Data collection and shooting in the field space are carried out through panoramic scanning and high-precision photography. Relevant data and graphics are matched and operated by algorithms to form a digital three-dimensional spatial model of the exhibition. Therefore, it is consistent with the actual scene of the exhibition.

**FIGURE 4:**
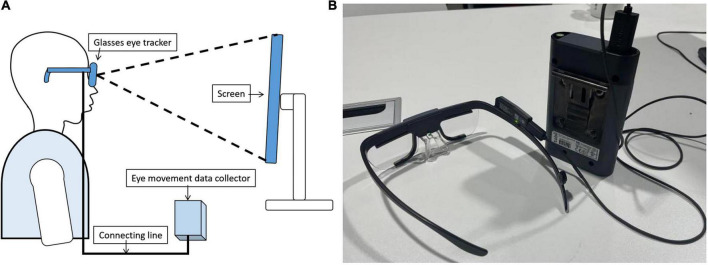
Schematic diagram of the experimental process and experimental equipment: **(A)** schematic diagram of the experimental process; **(B)** experimental equipment (brand name of the equipment: Tobii Pro Glasses Eye Tracker).

We provide a visual assessment of the visitor’s learning experience around three elements. The three contents are the content layout of the exhibition, the spatial form of the exhibition, and the display method of the exhibits. An observer’s eye movements are often informative about how the observer interacts with and processes a visual stimulus (Bylinskii et al., 2015). We obtained eye movement data of visitors by using the Tobii Pro Glasses eye tracker device (Figure 4). Visitors are the 14 subjects who participated in our experiment. We mainly collect visitors’ eye tracking data, hotspot data, Area of Interest (AOI) data, etc. We will quantitatively analyze the collected data on the Tobii Pro Lab eye movement data analysis platform. Finally, we combined questionnaires and interviews to comprehensively form a mapping evaluation of the display content, space and exhibits.

**FIGURE 5:**
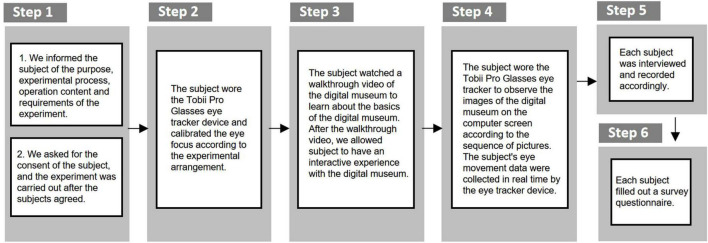
Flowchart of the experiment.

There were six steps in the experiment process (Figure 5). In the first step, we informed the subjects of the purpose, experimental process, operation content, and requirements of the experiment. We asked for the consent of the subject, and the experiment was carried out after the subjects agreed. In the second step, the subject wore the Tobii Pro Glasses eye tracker device and calibrated the eye focus according to the experimental arrangement. In the third step, we demonstrated on the computer screen the automatic roaming screen recording video of the online digital exhibition of Nanjing Museum-Yutang Jiaji Exhibition. After the roaming screen recording video was over, we allowed participants to have an interactive experience of this digital museum. This process was to let the subjects know the basic information of the digital museum. In the fourth step, we put the screenshots of the spatial scenes of each exhibition area in one picture in advance. This picture was put on the computer screen during the experiment. The participants watched the large picture on the computer screen. The purpose of this step was to test which exhibition hall’s display space form is more interesting to visitors in the case of the comparison of all exhibition halls. Then, we put the representative screenshots of showcases in the exhibition area in a picture in advance. In the experiment, this picture was put on the computer screen. We asked participants to view this large image on a computer screen. The experimental purpose of this step was to test which showcase in the exhibition was more interesting to the visitor. In the whole process of the fourth step, we recorded various eye movement indicators in the Tobii Pro Lab eye movement data analysis platform. In the fifth step, we conducted an interview and exchange of subjective feelings for each participant, and make corresponding written records. In the sixth step, we conducted a questionnaire survey on the participants. The purpose of interviews and questionnaires was to form qualitative research on this issue, to make up for the deficiencies of quantitative research, and to explain and corroborate the results of quantitative research.

## Analysis of experimental results

**FIGURE 6:**
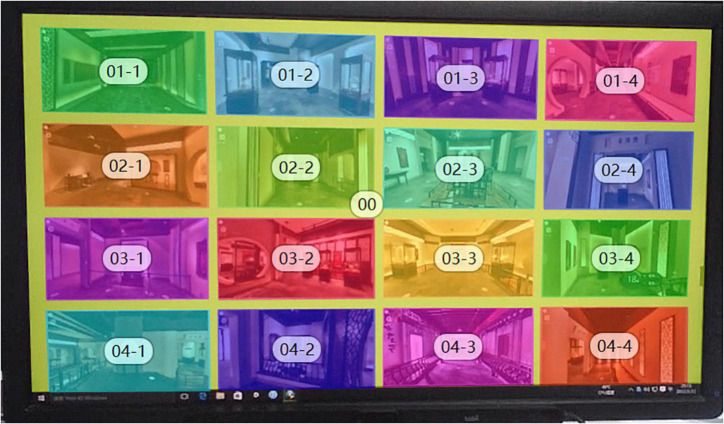
Schematic diagram of AOI area division of digital museum space test map.

**FIGURE 7:**
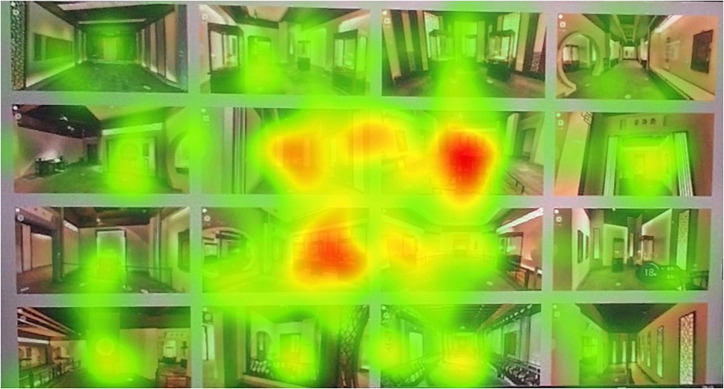
Total heat map of subject’s fixation.

After the experiment, we imported the data recorded in the Tobii Pro Glasses eye tracker device into Tobii Pro Lab for relevant analysis of the eye tracking data. First, the total space scene graph of our online digital museum is divided into areas of interest. Because the style of each space in this digital exhibition hall is consistent. Therefore, these spaces are all basically the same in terms of main materials, space tone, space lighting intensity, etc. The scenes in each space did not cause the subjects’ visual attention due to the obvious differences in styles. In addition to the total AOI-00 for the entire image, we marked a total of 16 scene AOI regions (Figure 6). The AOI is the area where the researcher is interested in the gaze of the test subject (Yao et al., 2011). Because the subjects have already experienced this digital museum in the early stage of the experiment. As a result, the subjects had a basic understanding of the narratives of these spaces. On this basis, we explore which exhibition hall spatial forms the subjects are more interested in. We performed eye-tracking data and image analysis for all subjects from the time they began to fixate on this image to around 30 s. The heat map can most intuitively reflect the eye gaze of the subjects during the experiment. The heat map can be used to find the visual objects that can attract the user’s attention the most, and compare the strong and weak relationships of each visual object to attract the user’s attention (Lu et al., 2015). We obtained the overall heat map by comprehensively overlaying the heat maps of the 14 subjects participating in the test (Figure 7). This overall heat map was analyzed by Tobii Pro Lab. The overall heat map analysis was performed because we found that each subject focused on something different when testing. The heat map of any subject can only represent the subject’s own eye movement data. We cannot analyze and judge the eye movement of the overall subjects through the analysis of individual eye movement data. By analyzing the total heat map of the subjects, we can find that the subjects pay the most attention to the exhibition hall space in the central area of the picture. The red areas in the picture are mainly concentrated in the AOI02-3, AOI03-2, AOI02-2, and AOI03-3 areas. The area with darker red indicates that the subject pays more attention to this area. The area with the most concentrated and visible red is AOI02-3. This shows that this area is the most concerned by the subjects. Because there is no complicated and difficult text information in these figures. And they are all space scenes with intuitive experience. Therefore, there is no incomprehensible visual stop condition. The green area is the area that subjects pay less attention to. An area without color indicates that the area did not attract the subjects’ obvious attention. In the picture, the green color concentration of AOI01-4 and AOI03-4 is not high, and the color is relatively light. Therefore, we can preliminarily judge that the form of exhibition hall space in AOI01-4 and AOI03-4 areas may need to be further optimized.

**FIGURE 8:**
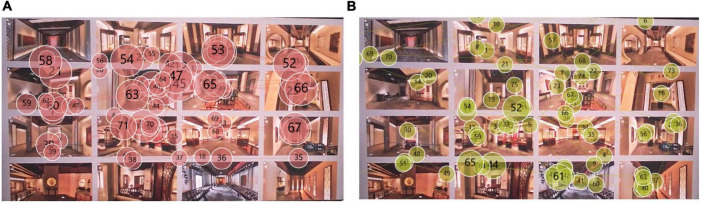
Subject’s gaze trajectory: **(A)** subject 1’s gaze trajectory; **(B)** subject 2’s gaze trajectory.

The analysis of the total heat map can tell us the overall concern of the spatial form. But the total heat map cannot show us more eye movement details. The eye movement track is an intuitive reflection of the gaze sequence and gaze degree of the subject’s eyes when viewing the picture. We can gain a deeper understanding of which parts of the exhibition hall space form are more able to attract the attention of the subjects through the analysis of the eye-tracking diagram. By analyzing each subject’s gaze trajectory, we can see which exhibition hall first caught the subject’s attention, and which exhibition halls the subject stared at for longer. The smaller the serial number in the circle, the first place the subject pays attention to, and the larger the need is, the later the subject pays attention to this place. For example, by observing the eye-tracking diagram of subject 1, we can find that the subjects’ attention is mainly concentrated in the vicinity of AOI02-2, AOI03-2, and AOI02-3 (Figure 8A). Most of the small and large circles overlap here. This shows that the spatial form of these areas has a certain attractiveness. By observing the eye-tracking diagram of subject No. 2, we can find that some small-numbered fixation points appear in the AOI01-2 area (Figure 8B), but no sustained attention is formed here. Therefore, it is possible to continue to improve the richness of the spatial sense of form.

The Tobii Pro Glasses eye tracker stored each subject’s eye movement data into the device. After the experiment, we removed the memory card from the device. We imported this raw information recorded on the memory card into the computer. We imported these raw information into the software platform system of the Tobii Pro Glasses eye tracker for analysis. After we imported these experimental data into the Tobii Pro Lab eye tracking data analysis platform, a series of indicators and comparison data were generated. We selected these data to build an experimental dataset. The data in our data set mainly counted the AOI data of the subjects. The data included time to first fixation, duration of first fixation, average duration of fixation, number of fixations, and total duration of fixation. We can further accurately analyze the attractiveness of each exhibition hall space to the subjects through the eye movement statistics of 14 subjects in 16 areas. Time to first fixation in AOI refers to the total time it takes from the presentation of the stimulus material to the start of fixation on an AOI or group of interest areas (Yan and Bai, 2018). The shorter the time, the easier it is to attract the attention of the subjects. From the following Tables 2, 3, we can find that the subject with the shortest first fixation time is AOI03-2. Its average value is 3.38. Therefore, the exhibition hall space in this area is most able to attract the attention of visitors. The interface with the shortest first fixation time of the subjects was AOI01-4. This shows that this space cannot attract the attention of visitors in the short term, and there is a possibility of further improvement. Duration of first fixation in AOI can illustrate the continuation of a space after attracting the attention of the subjects. We can find that the longest duration of first fixation is AOI04-3. Its average value is 0.42. The one with the shortest duration of first fixation was AOI03-1. Its average value is 0.17. The average duration of fixation in AOI indicator indicates the average fixation time of the subjects in the AOI area. The longer the time, the more likely the AOI area is to attract the subjects. The average value for AOI04-3 was 0.34. This is the largest value in each AOI area. This shows that AOI04-3 can arouse the continuous attention of the subjects. This exhibition hall has certain spatial characteristics. AOI01-1 is the smallest value in each AOI area. The average value of AOI01-1 was 0.21. This shows the lack of spatial form features in this showroom space. By comparing the number of fixations in AOI, we can find that the average value of AOI02-3 is 9.50. The average value of the AOI03-2 area is 9.21. This shows that these two areas have better spatial representation and can attract the attention of the subjects. By comparing the total duration of fixation in AOI, we can find that the average value of AOI02-3 is 3.5. The subjects’ attention to this area accounted for 8.67% of the total attention share. This is the best attention of any showroom space. Based on the above analysis charts and data, we can compare the averages of the indicators in each AOI area. AOI regions that fail to achieve mean alignment can be regarded as spatial regions that require further optimization.

**TABLE 2:** Eye movement statistics of subjects from AOI 01-1 to AOI 02-4.

		01-1	01-2	01-3	01-4	02-1	02-2	02-3	02-4
Time to first fixation in AOI	Average	9.36	6.63	10.09	11.00	7.75	4.25	3.64	9.05
	Variance	97.97	58.84	62.91	60.38	53.47	27.89	12.23	43.86
	SD (*n*−1)	9.90	7.67	7.93	7.77	7.31	5.28	3.50	6.62
Duration of first fixation in AOI	Average	0.22	0.24	0.27	0.31	0.25	0.22	0.30	0.24
	Variance	0.01	0.02	0.02	0.04	0.03	0.04	0.02	0.01
	SD (*n*−1)	0.11	0.15	0.15	0.19	0.17	0.21	0.15	0.11
Average duration of fixation in AOI	Average	0.21	0.22	0.26	0.32	0.26	0.23	0.33	0.26
	Variance	0.00	0.01	0.02	0.02	0.00	0.01	0.03	0.01
	SD (*n*−1)	0.06	0.09	0.13	0.15	0.07	0.09	0.16	0.11
Number of fixations in AOI	Average	2.25	4.50	3.64	3.00	3.46	8.29	9.50	3.75
	Variance	1.48	7.55	9.25	2.50	4.77	28.84	61.35	6.75
	SD (*n*−1)	1.22	2.75	3.04	1.58	2.18	5.37	7.83	2.60
Total duration of fixation in AOI	Average	0.46	1.03	0.91	0.96	0.94	2.20	3.50	1.04
	Share of total time (%)	0.98	2.18	1.78	1.53	2.15	5.44	8.67	2.21
	Variance	0.08	0.58	0.53	0.48	0.48	4.41	16.37	0.87
	SD (*n*−1)	0.28	0.76	0.73	0.70	0.69	2.10	4.05	0.93

**TABLE 3:** Eye movement statistics of subjects from AOI 03-1 to AOI 04-4.

		03-1	03-2	03-3	03-4	04-1	04-2	04-3	04-4
Time to first fixation in AOI	Average	8.27	3.38	4.01	10.76	7.12	10.15	8.22	9.84
	Variance	68.83	26.53	16.31	43.28	34.42	69.75	41.01	60.16
	SD (*n*−1)	8.30	5.15	4.04	6.58	5.87	8.35	6.40	7.76
Duration of first fixation in AOI	Average	0.17	0.21	0.22	0.27	0.30	0.22	0.42	0.21
	Variance	0.00	0.01	0.01	0.01	0.04	0.01	0.16	0.01
	SD (*n*−1)	0.05	0.10	0.09	0.12	0.20	0.09	0.40	0.10
Average duration of fixation in AOI	Average	0.25	0.26	0.23	0.27	0.29	0.24	0.34	0.22
	Variance	0.01	0.01	0.00	0.03	0.02	0.00	0.07	0.01
	SD (*n*−1)	0.08	0.08	0.05	0.17	0.14	0.07	0.26	0.09
Number of fixations in AOI	Average	2.70	9.21	6.14	3.30	5.00	4.31	4.92	3.20
	Variance	1.34	34.64	12.59	2.23	17.75	15.06	8.91	9.96
	SD (*n*−1)	1.16	5.89	3.55	1.49	4.21	3.88	2.99	3.16
Total duration of fixation in AOI	Average	0.74	2.33	1.53	0.79	1.56	1.00	1.44	0.66
	Share of total time (%)	1.31	5.78	3.78	1.40	2.48	2.31	3.30	1.18
	Variance	0.26	1.92	1.07	0.17	3.02	0.71	0.99	0.28
	SD (*n*−1)	0.51	1.39	1.04	0.41	1.74	0.84	1.00	0.53

**FIGURE 9:**
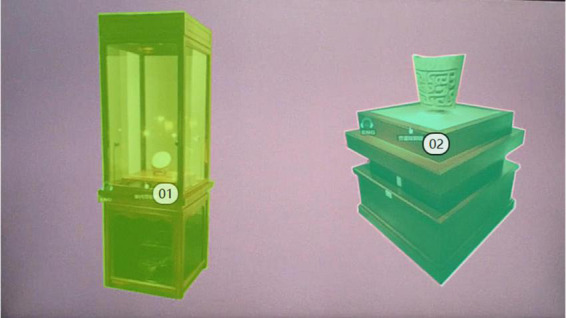
Schematic diagram of AOI area division of digital museum showcase test map.

**FIGURE 10:**
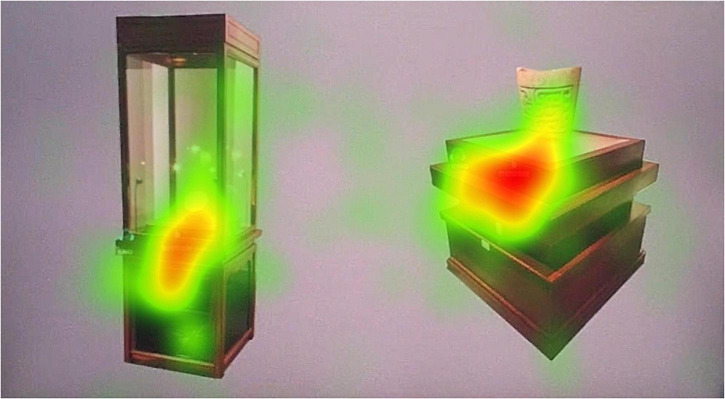
Total heat map of subject’s fixation.

**FIGURE 11:**
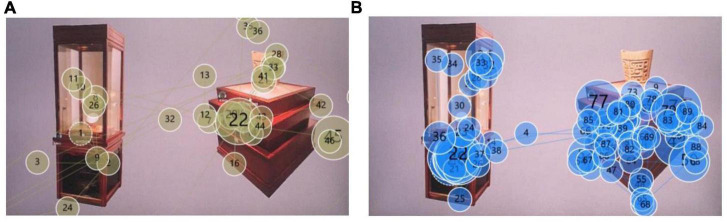
Subject’s gaze trajectory: **(A)** subject 2’s gaze trajectory; **(B)** subject 3’s gaze trajectory.

The way the exhibits are displayed in the museum is very important to the evaluation of the museum display. The showcase is an important embodiment of the way the exhibits are displayed. We selected the most representative two types of showcases in this digital museum for eye-tracking experimental analysis. The two showcases are the same in style. We divided the AOI according to the shape of the two booths in Tobii Pro Lab (Figure 9). The overall heat map was obtained by image overlay analysis of the gaze heat maps of 14 subjects. From the overall heat map we can see Figure 10 that the area of AOI-02 produces a more pronounced red area. AOI-02 produces a larger area of red and a deeper degree of color than AOI-01. This shows that the subjects pay more attention to the booth display of AOI-02. The display form of AOI-02 is easier to attract the attention of the subjects. We can further discover the observation process of each subject’s display of exhibits through the subject’s trajectory map. After observation, it was found that most of the subjects produced more circles and denser in the AOI-02 area. This shows that the booth of AOI-02 has received more attention. For example, subject No. 2 focused on the AOI-02 area (Figure 11A). Obvious attention was formed in both the exhibits area and the booth area of the AOI-02 booth. However, the subject’s attention in the AOI-01 area was scattered, and the concentration was not high. This shows that the display method of AOI-01’s exhibits needs to be improved. In the AOI-02 area. Subject 3 had a more dense and uniform distribution of circles in the AOI-02 area (Figure 11B). But the circles are mostly concentrated on the concave and convex shape of the booth. This shows that although the booth of AOI-02 is easy to show a better display effect in the space. But in the later stage, we also need to consider how to highlight the exhibits and avoid the booth from attracting the attention of the audience too much.

Through the statistics of the subjects’ AOI data (Table 4), we can further accurately analyze the subjects’ eye movement and visual evaluation of the two types of booths. In the time to first fixation in AOI values, the average value of AOI-02 was lower. The average value for AOI-02 was 1.02. The average value for AOI-01 was 1.28. This data shows that subjects are more likely to pay attention to AOI-02. It will take longer to focus on AOI-01. The variance and SD (*n*−1) values of AOI-02 are also significantly lower than those of AOI-01. This shows that the numerical stability of AOI-02 is also very good. In the duration of first fixation in AOI value, the average value of AOI-01 was 0.31. The average value for AOI-02 was 0.20. This means that the subjects lasted longer in AOI-01 during the first attention process. However, the variance and SD (*n*−1) values of AOI-01 were significantly higher than those of AOI-02. This shows that the observed values of AOI-01 produced by the subjects have a relatively large degree of dispersion, and the values are unstable. In the average duration of fixation in AOI values, the average value of AOI-01 was 0.42. The average value for AOI-02 was 0.39. The average value for AOI-01 was slightly higher. However, the variance and SD (*n*−1) values of AOI-01 are higher than those of AOI-02. The performance of the two is basically the same in this regard. In the number of fixations in AOI value, the average value of AOI-02 is 26.86. This value is higher than AOI-01. Therefore, it can be judged that the subjects are paying more attention to AOI-02. In the value of total duration of fixation in AOI, the total attention time in AOI-02 accounted for 44.35%. The total attention time in AOI-01 accounted for 28.44%. These data comprehensively show that the way the exhibits of AOI-02 are displayed is more likely to attract the attention of the subjects from the perspective of eye-tracking engineering verification.

**TABLE 4:** The eye movement data statistics of the subjects in the AOI area.

		01	02	Average	Median
Time to first fixation in AOI	Average	1.28	1.02	1.15	1.15
	Variance	6.21	0.94	1.33	1.33
	SD (*n***−**1)	2.49	0.97	1.15	1.15
Duration of first fixation in AOI	Average	0.31	0.20	0.25	0.25
	Count	14	14		
	Variance	0.14	0.02	0.05	0.05
	SD (*n***−**1)	0.38	0.14	0.22	0.22
Average duration of fixation in AOI	Average	0.42	0.39	0.40	0.40
	Variance	0.05	0.04	0.04	0.04
	SD (*n***−**1)	0.22	0.20	0.19	0.19
Number of fixations in AOI	Average	22.07	26.86	24.46	24.46
	Variance	177.30	134.75	62.79	62.79
	SD (*n***−**1)	13.32	11.61	7.92	7.92
Total duration of fixation in AOI	Average	8.58	10.39	9.48	9.48
	Share of total time (%)	45.22	54.78		
	Variance	28.44	44.35	10.81	10.81
	SD (*n***−**1)	5.33	6.66	3.29	3.29

## Experimental discussion

After the eye-tracking experiment, we conducted interviews and questionnaires on the subjects. When asked if the subjects could remember the route of the roaming scene of the digital museum, more than half of the subjects held a negative or negative attitude. Most of the subjects indicated that they could not remember the route of the roaming scene or had a vague memory of the route of the roaming scene. This shows that the spatial narrative and streamline arrangement of the museum are not clear enough. When asked how the subjects could remember the scenes of several exhibition areas of the digital museum, five subjects indicated that the number of memorized exhibition areas was 7 or less. Only four subjects could remember exactly the number of exhibition halls. This reflects from the side that the characteristics of each space in the digital museum are not obvious enough. The digital museum exhibition hall cannot form a clear and complete experience process in the memory of visitors. Five respondents said they had the deepest memory of the lounge space in the exhibition. Four respondents said that the “Qu Ping Xiang Nuan” exhibition hall was more impressive. The reason why these two exhibition halls are so impressive is basically because the space features are obvious and the space has a good sense of form. Four subjects had the deepest memory of the celadon sheep exhibits in the museum. The reason for the deep impression is not only related to the exhibits themselves, but also to the better way of display. Some respondents said that the characteristics of the current exhibition area are not clear enough, the display forms are not rich enough, and the interactive experience of visitors is not enough. These opinions are also consistent with the test results of the previous eye-tracking experiment, when the subjects’ focus was only concentrated in a few exhibition halls. This shows that most of the exhibition halls need to continue to improve and optimize in terms of space form characteristics and exhibit display methods. The analysis results of the questionnaire survey show that all respondents strongly agree or basically agree that digital museums need to have a clear layout of exhibition content. All respondents strongly agree or basically agree that digital museums need to have a variety of display space forms. More than 90% of the respondents strongly agree or basically agree that digital museums need creative ways to display exhibits. These data show that the subjects attach great importance to the layout of the exhibition content, the form of the exhibition space and the way of displaying the exhibits in the digital museum. More than 68% of the respondents strongly agree or basically agree that the evaluation of the information experience of digital museum visitors should focus on the layout of exhibition content. More than 90% of the respondents strongly agree or basically agree that the evaluation of the information experience of digital museum viewers should focus on the display space form. All the respondents strongly agree or basically agree that the evaluation of the information experience of digital museum visitors should focus on the display method of exhibits. Therefore, it is in line with most people’s evaluation criteria to evaluate the information experience of digital museum visitors from the layout of exhibition content, the form of display space and the way of exhibits. More than 84% of the respondents strongly agree or basically agree that the exhibition content layout of the digital museum in this experiment is reasonable. More than 76% of the respondents strongly agree or basically agree that the display space form of the digital museum in this experiment is reasonable. More than 69% of the respondents strongly agree or basically agree that the display method of the exhibits in the digital museum of this experiment is reasonable. From the perspective of the subjects’ feedback, it can be seen that the subjects are more satisfied with the information experience of the digital museum viewers in this experiment.

**FIGURE 12:**
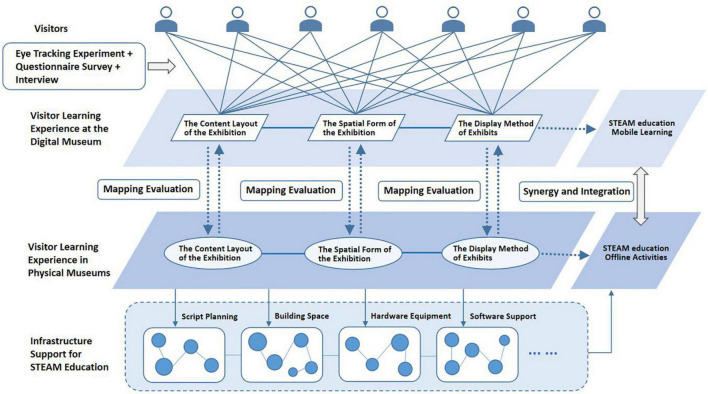
Model diagram of multi-dimensional learning experience evaluation framework for visitors in digital museums.

The visual evaluation of the learning experience of digital museum visitors should focus on the layout of exhibition content, the form of exhibition space, and the way of display of exhibits (Figure 12). Visitors can form a visual evaluation of the learning experience through the experience of the digital museum. This evaluation can directly form a mapping relationship to these three aspects of the physical museum. And this kind of evaluation can affect the infrastructure support of the physical museum, such as large script planning, architectural space, hardware equipment, and software support. We use the Tobii Pro Glasses eye tracker device and the Tobii Pro Lab system to analyze the visitor’s eye tracking data, hot spot data, fixation time data, etc. These data can reflect the learning experience of visitors to the museum’s spatial narrative, spatial form, and exhibits. Combining questionnaires and interviews, we can conduct a comprehensive evaluation of visitor learning experiences in digital museum.

Compared with the previous related research work, this study has mainly improved in three aspects. First, we focus on improving the design quality of the digital museum and the satisfaction of visitors with the accurate visual evaluation of eye trackers. In previous related researches, digital museum design was dominated by technology (Lui et al., 2007). It also conducted research on digital museum design based on the accumulation and summary of design experience (Zhai and Zhang, 2016). Second, in the process of conventional design practice, digital museums are often regarded as online publicity tools or supplements of display functions of physical museums. In this study, we try to explore the use of digital museums as an important carrier for visual evaluation to promote the improvement of the overall display design of museums. Third, the exploration of visual evaluation methods using eye trackers has been successfully applied to clothing design (Li et al., 2021), product design (Zhang and Zhu, 2019), and landscape design (Zhao et al., 2020). However, the research of this evaluation method in the field of digital museum display design is still insufficient. We try to establish an effective path for evaluation from the layout of exhibition content, the form of exhibition space, and the way of display of exhibits. And we promote the improvement of STEAM education in digital museums through this research.

## Conclusion

From the survey of museology practice, the combination of digital museum construction and STEAM education is insufficient. Although the visitor experience has been paid attention to in the research of digital museums. However, the survey shows that the research and practice of visitors’ learning experience in digital museums needs to be further improved. Experiential learning for digital museums lacks a more multivariate evaluation method. Due to the impact of COVID-19 and the improvement of global digitalization and Internet technology, the current experience of digital museums is mainly a mobile experience through mobile terminals or computer screens. These tools are readily available and easy to operate on a daily basis. Therefore, mobile terminals and computers affect the relationship between digital museums and audiences in such intensive and powerful way. These tools enable visitors to obtain relevant museum information mainly through the visual experience. Effective dissemination of visual information can help digital museums conduct educational activities more efficiently. A visitor’s visual evaluation of a digital museum can reflect the depth of the visitor’s learning experience. The digital museum learning experience based on the visual evaluation of visitors needs to pay attention to the content layout of the exhibition, the space scene displayed, and the display method of the exhibits. Based on these evaluation dimensions, visitors can develop interdisciplinary and contextualized STEAM educational activities. The process of visual evaluation needs to be based on the basic framework model of “visitor layer – digital museum layer – physical museum layer.” The eye movement engineering experiment can quantitatively and accurately evaluate the multi-dimensional learning experience of visitors. The eye movement trajectories, visual hotspots, and eye movement data of visitors in the digital museum have positive significance for the reference of visual evaluation. Combined with questionnaires and interviews, we can comprehensively evaluate the learning experience of visitors in the digital museum. STEAM education can rely on digital museums to conduct online learning and exploration models. A digital museum can also become a test body for the learning experience of a physical museum. It can help physical museums complete simulation experiments and performance tests under various hypothetical conditions. The learning experience of digital museums can also enrich and improve the STEAM educational functions in offline museums. In the future, we can form a more comprehensive and systematic method by comparing the differences in the visual evaluation of public education experiences in different digital museums based on research objects of different themes and scales.

## Data availability statement

The raw data supporting the conclusions of this article will be made available by the authors, without undue reservation.

## Author contributions

XZ and JH: conceptualization, methodology, validation, writing—original draft preparation, and writing—review and editing. XZ: formal analysis. JH: supervision and project administration. Both authors read and agreed to the published version of the manuscript.
